# Soil microbiota promote the success of the perennial legume *Lupinus polyphyllus* more strongly in invasive than in native populations

**DOI:** 10.1093/aob/mcag067

**Published:** 2026-03-19

**Authors:** Annika Nylund, Aino Kalske, Seyed Abdollah Mousavi, Satu Ramula

**Affiliations:** Department of Biology, University of Turku, Turku 20014, Finland; Department of Biology, University of Turku, Turku 20014, Finland; Department of Biology, University of Turku, Turku 20014, Finland; Department of Biology, University of Turku, Turku 20014, Finland

**Keywords:** Adaptation, allelopathy, enemy release, enhanced mutualism, invasive plant, *Lupinus polyphyllus*, plant–soil microbe interactions, rhizobia

## Abstract

**Background and Aims:**

Geographical variation in soil microbial communities can give rise to differences in the performance and competitive abilities of plants between the invasive and native ranges, potentially due to the presence of more efficient mutualists or release from pathogens in the invasive range.

**Methods:**

We investigated how microbial inoculation with soil taken from invasive and native sites of the legume *Lupinus polyphyllus* affects the performance of plants of both origins and their below-ground bacterial communities. We also exposed a co-occurring herb from the invasive range to a substrate that had previously been occupied by inoculated and non-inoculated *L. polyphyllus* to assess whether soil microbes mediate its competitive allelopathic effects.

**Key Results:**

We found that for plants of invasive origin, inoculation with soil from the invasive range enhanced performance more than inoculum from the native range. For plants of native origin, instead, performance was facilitated equally by the microbiota from both ranges. Substrate that had been occupied by *L. polyphyllus* did not inhibit the germination of the co-occurring herb. Differences in the composition of the below-ground bacterial communities between plants grown in soil inocula from invasive and native sites at the family level were minor.

**Conclusions:**

Our results advance the mechanistic understanding of plant invasions, suggesting that soil mutualists in the invasive range may facilitate invasion success as proposed by the enhanced mutualism hypothesis. Alternatively, greater performance of invasive plants with soil microbes from their own range could reflect other adaptations either in plants or soil microbiota, or in both.

## INTRODUCTION

An important element in plant invasions is the interaction between plants and soil microbiota, which includes both soil-borne antagonists (e.g. pathogens and parasites) as well as mutualists (e.g. mycorrhizal fungi and nitrogen-fixing bacteria) (Reinhart and Callaway, 2006). Differences in soil microbial communities between the native and introduced range associated with a given plant species may result in altered interactions that can potentially lead to differences in plant performance traits, as well as playing a prominent role in how plant invasions proceed (Reinhart and Callaway, 2006; Dawson and Schrama, 2016).

One potential explanation for the differences in plant performance traits between native and invasive populations could be the presence of more efficient local soil mutualists in novel areas compared with the native range, as predicted by the enhanced mutualism hypothesis (Reinhart and Callaway, 2006; Sun and He, 2010; Dawson and Schrama, 2016). For instance, for the annual weed *Conyza canadensis*, differences in mycorrhizal associations between ranges may contribute to the larger size and better reproductive capacity of individuals in the introduced range (Sheng *et al.*, 2022). The absence of natural enemies, such as soil pathogens, can also enhance plant performance in the introduced range, as postulated by the enemy release hypothesis (Keane and Crawley, 2002; Zhao *et al.*, 2020). Conversely, introduced plant populations may also encounter the absence of compatible soil mutualists in the novel area (Catford *et al.*, 2009), which may be a barrier for invasions and cause rapid evolution in introduced populations towards reduced dependence on mutualists (Seifert *et al.*, 2009; Simonsen *et al.*, 2017; Kalske *et al.*, 2022).

A widespread type of mutualistic soil microbe is the group of bacteria known as rhizobia, which induce the formation of root nodules and reduce atmospheric nitrogen (N_2_) to a form that can be assimilated by plants (Andrews *et al.*, 2013). Symbiosis with rhizobia benefits the host plant by, for example, increasing the nitrogen content of its leaves and seeds, which may improve a plant’s germination, growth, herbivore defence and competitive ability (Thrall *et al.*, 2011; Adams *et al.*, 2016; Kalske *et al.*, 2022; Mathesius, 2022). Due to the fitness benefits of rhizobia, plants can be highly dependent on these mutualistic interactions (Simonsen *et al.*, 2017; Mathesius, 2022). Moreover, previous studies have observed differences in the symbiotic effectiveness of different rhizobial strains (Thrall *et al.*, 2011; Ramula *et al.*, 2023), which may constrain the invasion success of introduced plant species (Simonsen *et al.*, 2017). Nonetheless, invasive plants are often capable of adapting to local rhizobia in the new area. For instance, legumes in the tribe Genisteae, including lupins (*Lupinus*), are known to associate with at least seven rhizobial genera (Stępkowski *et al.*, 2018). The ability of invasive plants (and legumes in particular) to form a symbiosis with a broad variety of rhizobial strains may be an important contributor to rapid establishment and expansion in novel areas (Klock *et al.*, 2015; Harrison *et al.*, 2018).

Another way that soil microbiota can affect plant performance is by influencing plant competitive interactions, particularly through mediating allelopathic effects between competing plants (Cipollini *et al.*, 2012). Allelopathy refers to a mechanism by which a plant releases allelochemicals that may interfere with the performance of co-occurring plant species, for example by impairing their growth or germination (Zhang *et al.*, 2021). Invasive plants commonly affect their local ecosystem via allelopathy, which can be more disruptive than the allelopathic effects of other co-occurring native plant species, suggesting that this form of competition may have an important role in plant invasions (Kalisz *et al.*, 2021; Zhang *et al.*, 2021). Soil microbes have been found to stimulate the production of allelochemicals or increase their toxicity (Cipollini *et al.*, 2012). However, soil microbiota may also reduce allelopathic effects through the degradation of allelochemicals released into the soil (Cipollini *et al.*, 2012; Li *et al.*, 2015).

Garden lupin, *Lupinus polyphyllus* (Fabaceae), is one of the worst plant invaders in Europe and is considered invasive also in New Zealand, Chile, eastern North America, Japan, west Siberia and southern Australia (Eckstein *et al.*, 2023). It is a short-lived perennial herb native to western North America, and was introduced to Europe and Finland in the 1800s (Eckstein *et al.*, 2023). In northern Europe, the species inhabits road verges and meadows, whereas in its native range it thrives in moist habitats along streams (Eckstein *et al.*, 2023). In Finland, it forms symbioses with different rhizobial strains of *Bradyrhizobium* (Ramula *et al.*, 2023), and native populations of the species are able to associate with rhizobia outside of their own range as well (Kalske *et al.*, 2022). Previous work demonstrated that microbial inoculation with soil from sites where *L. polyphyllus* is invasive benefited plants of both invasive and native origins, indicating that the soil microbiota from the invasive range had a net positive effect on both invasive and native populations of the species (Kalske *et al.*, 2022). In other words, the presence of rhizobia and other soil mutualists overrode any possible negative effects of soil pathogens in the invasive range. However, that study did not examine the effects of native soil microbiota, which may be more likely to harbour specialist soil pathogens. Moreover, *L. polyphyllus* is known to exert allelopathic effects on co-occurring native plant species that reduce their germination (Kalske *et al.*, 2023), but the role of soil microbiota in this phenomenon remains unclear.

Here, we investigated how microbial inoculation with soil from invasive (Finnish, FI) and native (North American, US) ranges of *L. polyphyllus* affects the performance of plants from both origins and their below-ground bacterial communities. We also explored whether soil microbes from the invasive range modify the potential allelopathic effects of the species on a co-occurring perennial herb (dandelion, *Taraxacum* sp.). We hypothesized that invasive plants of *L. polyphyllus* would benefit more from invasive soil microbiota than native soil microbiota. A stronger positive effect of soil microbes in the invasive range could be due to more efficient mutualists that enhance plant growth (the enhanced mutualism hypothesis; Sun and He, 2010) and/or fewer pathogens that reduce plant performance (the enemy release hypothesis; Reinhart and Callaway, 2006). Secondly, as native (US) plant populations may be more adapted to their local soil microbiota and/or invasive (FI) populations may have evolved to be less dependent on soil mutualists (Kalske *et al.*, 2022), we expected that a substrate inoculated with soil microbes from the native (US) range would increase the performance of plants of native origin to a greater degree than for plants of invasive (FI) origin. Finally, because local soil microbes of invasive plant species may gradually start to reduce the negative allelopathic effects of invaders (Li *et al.*, 2015), we predicted that the presence of soil microbes associated with *L. polyphyllus* would alleviate its allelopathic effects on a co-occurring perennial herb compared with soil without lupin-associated microbes.

## MATERIALS AND METHODS

We collected seeds from three invasive Finnish (FI) and three native North American (US) populations in July 2018 (Supplementary Data Table S1) by haphazardly choosing 20 maternal plants that were at least 2 m apart in each population. The native populations inhabit lower latitudes than the invasive populations, but the mean annual temperatures between them are similar (Ramula and Kalske, 2020). All Finnish populations were at least 10 years old (S. Ramula, pers. obs.).

We sampled soil for the experiment from the same six populations where the seeds had been collected. We collected 0.3 L of soil from the rhizosphere of *L. polyphyllus* at a depth of 10 cm, and sterilized the shovel in commercial bleach between sites to prevent cross-contamination. Collection of soils was done in late January 2023 by sampling US soil under snow, and in early April 2023 when the ground had thawed in Finland. We placed the samples from both origins in zip-lock bags, transported them to the laboratory as soon as possible, and stored them at +4 °C until the experiment (for ∼3 months for the US soils and 1 month for the FI soils). For logistic reasons, transport time was longer for the US than for FI soils.

### Experimental setups

At the end of April 2023, we surface-sterilized 80 seeds per population for the experiment as in (Ramula *et al.*, 2023). For each seed, we nicked the seed coat with a scalpel to improve germination; the seeds were then placed on a moist paper towel in a plastic-covered aluminium dish and kept at room temperature with ambient light. Six days after sowing, we planted seedlings in 1-L pots in a 1:1 mix of commercial sand and vermiculite. Two days later we inoculated each pot with 10 mL of autoclaved or intact soil inoculant from one of the six populations (Supplementary Data Table S1). We prepared the soil inoculants by mixing 200 g of soil in 1 L of water (Lau and Suwa, 2016). Half of the inoculant from each population was autoclaved at 120 °C, 1 bar, for 20 min to reduce microbes for an experimental control. The growth medium did not contain meaningful amounts of rhizobia (Supplementary Data Methods S1) and nodulation was greatly reduced in the autoclaved treatment (see Results section). Microbial activity tests on tryptone–yeast agar plates showed that the US and FI intact soil inocula contained comparable amounts of living microbes (Supplementary Data Methods S1).

The inoculation of plants was fully reciprocal for both intact and autoclaved inoculants. We repeated each combination of plant population (three US and three FI) by soil inoculum site (three US and three FI) by soil inoculum treatment (autoclaved, intact) four times (6 plant populations × 6 inoculum sites × 2 inoculum treatments × 4, altogether 288 plants). We arranged the pots in a greenhouse in four blocks (randomized within blocks and spaced ∼ 10 cm apart). We used a bottom watering tray for each pot to prevent the transfer of soil microbes between pots and grew the plants in ambient light and temperature. The surface of each pot was covered by a 1-cm layer of lightweight expanded clay aggregate (LECA) to prevent cross-contamination. We watered the plants regularly with filtered tap water (Sawyer Mini Water Filter, Sawyer Products, Safety Harbor, FL, USA) and fertilized them twice during the 2-month experiment with a plant growth nutrient solution (40 mL per pot), which is recommended for legumes grown in sand (p. 91 in Howieson and Dilworth, 2016).

We recorded plant height 19 d after sowing for a measure of initial plant size. We then allowed the plants to grow for 6 more weeks and measured their height, number of leaves and number of leaflets per leaf at the end of June. We also estimated the photosynthetic capacity of the plants with a portable chlorophyll meter (SPAD-502 Plus, Konica Minolta, Tokyo, Japan) by taking readings from three different fully grown leaflets per plant. The SPAD value (arbitrary unit) estimates the relative amount of chlorophyll present in the leaf, with higher values generally indicating healthier plants. Finally, we collected substrate samples from all pots (depth of 3–5 cm) to characterize below-ground bacterial communities. We then harvested above-ground biomass, roots and nodules. We washed the roots and when still fresh we counted the number of nodules and investigated the activity of up to five randomly chosen nodules on each plant by cutting them open and visually inspecting the colour. Nodules with a red interior were considered active (fixing nitrogen) and nodules with brown or white interior inactive (Howieson and Dilworth, 2016). We dried all harvested material at 60 °C for 4 d and weighed it for a measure of final plant size (roots, shoots and nodules separately).

To investigate whether soil microbiota from the invasive sites of *L. polyphyllus* mediate the allelopathic effects of lupin on a co-occurring perennial herb, we conducted a common garden experiment based on a substrate conditioned in the greenhouse experiment (see above). Dandelion (*Taraxacum* sp.) is a common perennial weed that colonizes a broad range of habitats including roadsides, where it co-exists with *L. polyphyllus* (S. Ramula, pers. obs.). Both root and shoot leachates of *L. polyphyllus* inhibit the germination of *Taraxacum* seedlings on Petri dishes (Kalske *et al.*, 2023). We kept all of the substrate of the pots that were inoculated with the FI inoculant (144 pots, with 72 FI and 72 US plants grown in them) and planted five locally collected *Taraxacum* seeds in each pot at the end of June (primed pots). For controls, we planted five *Taraxacum* seeds in each of 52 pots filled with the same substrate without adding a field soil microbial inoculant or *L. polyphyllus* (unprimed pots). Due to biosecurity risks related to soil microbes from the native range if transferred to a common garden, the pots with the US soil inoculum were not included in this experiment. We placed the pots on the soil surface covered with a tarpaulin in a common garden and top-watered them manually when necessary. We recorded the number of *Taraxacum* seedlings after 2 and 5 weeks, and measured the diameter of three plants per pot (when possible) after 7 weeks.

### Below-ground bacterial communities

To characterize the bacterial communities of the soil inocula (six sites) and of the pots in the greenhouse experiment (288 pots), we extracted the genomic DNA of microorganisms from each sample with the NucleoSpin Soil kit (Macherey-Nagel GmbH Co. KG, Duren, Germany) following the steps provided by the manufacturer. We amplified DNA using primers targeting the v4 region of the 16S rRNA gene. We used a standard bioinformatic pipeline that included merging, filtering, trimming and clustering the reads into amplicon sequence variants (ASVs) as in Mousavi and Ramula (2024). Details of sampling, DNA extraction and bioinformatics are described in Supplementary Data Methods S2.

### Statistical analyses

We conducted statistical analyses in R version 4.3.1 (R Core Team, 2023). For plant total biomass (log-transformed), number of leaves (log-transformed), number of leaflets, chlorophyll concentration and root:shoot ratio (log-transformed), we used linear mixed-effect models (LMMs, lme4::lmer; Bates *et al.*, 2015) with plant origin (FI, US), soil inoculum origin (FI, US), soil inoculum treatment (autoclaved, intact) and all possible interactions between them as fixed explanatory variables. Initial plant height was included as a covariate because plants from invasive (FI) populations were taller than plants from native (US) populations at the beginning of the experiment (mean: FI, 3.95 cm; US, 1.98 cm). In each model, we included plant population and block as random explanatory variables because their variances were higher than that of the combination of soil population and block, and including all three caused convergence problems. The five response variables were analysed separately as there were no strong correlations between them (*r* < 0.69 for all). We excluded plant final height from the analyses because it correlated positively with total biomass (*r* = 0.86).

Prior to nodule trait analyses, we excluded all individuals inoculated with the autoclaved soil inoculant from the data because most of them (93.1 %) did not produce nodules (i.e. autoclaving reduced the amount of soil microbes, including nodule-forming rhizobia). For the number of nodules and nodule activity, we used generalized linear mixed-effect models (GLMMs, glmmTMB::glmmTMB; Brooks *et al.*, 2017) with plant origin, soil origin and an interaction between them as fixed explanatory variables. In addition, we used initial height as a covariate to account for differences in plant size. The models contained the same random explanatory variables as the previous models. For the number of nodules, we specified a negative binomial distribution with a log link function, while for nodule activity (measured as the number of red nodules out of up to five nodules per plant) we specified a binomial distribution with a logit link function. We excluded nodule biomass from the analyses due to its positive correlation with the number of nodules (*r* = 0.75).

To assess the allelopathic effect of *L. polyphyllus* on a co-occurring perennial herb, we conducted four linear fixed-effect models with the number of *Taraxacum* seedlings during the first measurement and mean seedling diameter (calculated from three seedlings per pot) at the end of the experiment as response variables. We first investigated whether earlier exposure to *L. polyphyllus*, irrespective of plant origin, affected the number or diameter (log-transformed) of *Taraxacum* seedlings (i.e. we compared the *L. polyphyllus*-primed pots with the control pots unprimed by plants or inoculum). We then focused on the *L. polyphyllus*-primed pots only and assessed whether the origin of the plant (FI, US), soil inoculum treatment (autoclaved, intact) or their interaction affected the number or the mean diameter of *Taraxacum* seedlings.

Model assumptions were verified from residual plots (for all models) and dispersion parameters (for GLMMs, DHARMa; Hartig, 2022). We transformed the data as necessary to meet the model assumptions (see above); all figures are based on back-transformed values. We used Tukey’s test to assess pairwise differences in mean values when any interactions had a significant effect on response variables (emmeans:emmeans; Lenth, 2023).

We investigated differences in the composition of the below-ground bacterial communities in the greenhouse experiment with a permutational multivariate analysis of variance (PERMANOVA, vegan::adonis2; Oksanen *et al.*, 2022) based on Bray–Curtis dissimilarities calculated from the relative abundances of bacterial ASVs. We used plant origin (FI, US), soil inoculum origin (FI, US), soil inoculum treatment (autoclaved, intact) and all possible interactions between them as fixed explanatory variables with 9999 permutations. The results were visualized with a non-metric multidimensional scaling (NMDS) based on two dimensions (stress = 0.17).

## RESULTS

### Plant performance

**Fig. 1.**
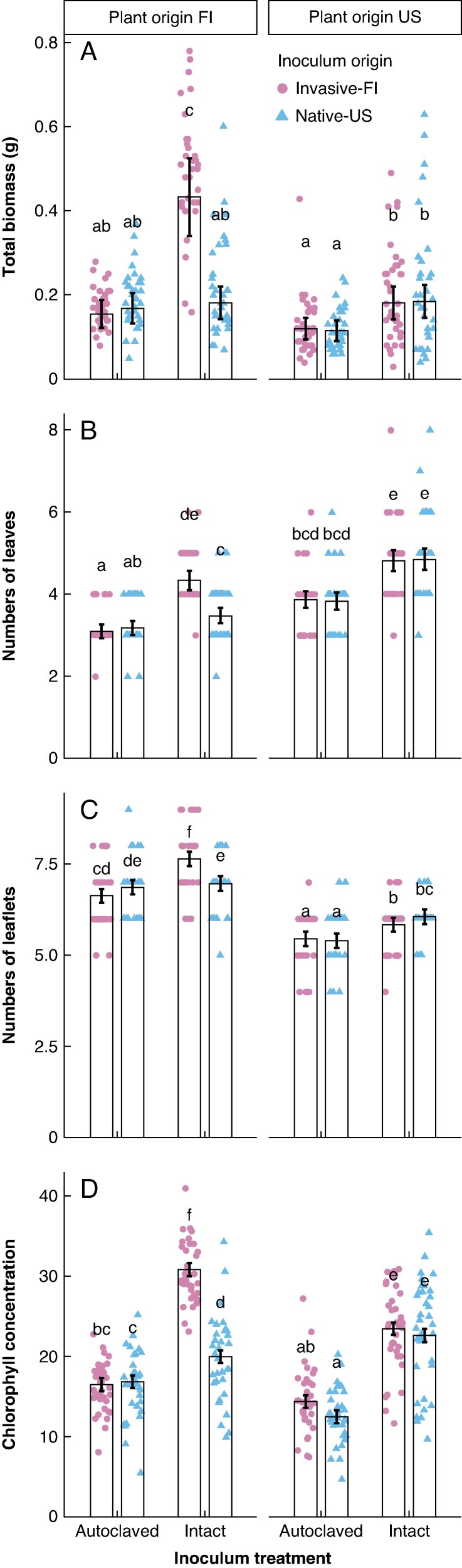
(A) Total biomass, (B) number of leaves, (C) number of leaflets and (D) chlorophyll concentration (SPAD value) of invasive (FI) and native (US) *Lupinus polyphyllus* grown in autoclaved and intact soil inoculum treatments (mean ± standard error). Points represent raw data points. Statistical differences are shown with different letters (*P* < 0.05, Tukey’s test).

The presence of soil microbes improved plant performance, but the effect depended on the combination of soil inoculum origin and plant origin for the four plant performance traits (three-way interactions for plant biomass, number of leaves, number of leaflets, chlorophyll concentration; Table 1). FI plants performed better in the intact FI soil inoculum treatment than in the autoclaved FI soil inoculum treatment for all four traits (biomass +179.4 %; number of leaves +42.8 %; number of leaflets +15.2 %; chlorophyll concentration +86.1 %; Table 1, Fig. 1A–D). For FI plants treated with US soil inoculum, the increase in performance in the intact inoculum treatment relative to the autoclaved one was smaller than with FI soil inoculum (biomass +7.7 %; number of leaves +9.3 %; chlorophyll concentration +19.2 %; Table 1, Fig. 1A, B, D). For US plants, the intact soil inoculum treatment improved plant performance relative to the autoclaved one to a similar degree regardless of soil inoculum origin (biomass: US soil +60.9 %, FI soil +50.8 %; number of leaves: US soil +26.3 %, FI soil +24.7 %; number of leaflets: US soil +12.3 %, FI soil +7.1 %; chlorophyll concentration: US soil +81.6 %, FI soil +63.2 %; Table 1, Fig. 1A–D). Finally, FI plants in the intact FI soil inoculum treatment outperformed those in the intact US soil inoculum treatment (biomass +139.2 %; number of leaves +25.8 %; number of leaflets +9.2 %; chlorophyll concentration +54.3 %), whereas for US plants none of these traits differed between the two origins of the intact soil inoculum (Table 1, Fig. 1A–D). In the intact US soil inoculum treatment, US plants outperformed FI plants for two traits (number of leaves +36 %; chlorophyll concentration +14.1 %; Table 1, Fig. 1B, D), while FI plants outperformed US plants for one trait (number of leaflets +15.2 %, Table 1, Fig. 1C). Initial plant height was positively associated with plant total biomass (intercept = −2.0662, slope = 0.0068) and the number of leaves (intercept = 0.0011, slope = 0.0005; Table 1).

**Table 1.** Results from linear mixed-effect models testing for differences in performance traits of *Lupinus polyphyllus* of different origins inoculated with soil from invasive (FI) and native (US) sites. Plant population and block were included as random effects in each model. Significant *P*-values (*P* < 0.05) are shown in bold; d.f. and d.d.f. denote the degrees of freedom in the numerator and denominator; NA, not applicable for generalized linear mixed-effect models.

Response variable	Explanatory variable	d.f., d.d.f.	*F*/χ^2^	*P*
Total biomass (g)	Plant origin (FI, US)	1, 4	1.63	0.268
	Soil inoculum origin (FI, US)	1, 272	19.25	**<0**.**001**
	Inoculum treatment (intact, autoclaved)	1, 272	115.68	**<0**.**001**
	Initial plant height (mm)	1, 277	11.33	**<0**.**001**
	Plant origin × soil inoculum origin	1, 272	17.19	**<0**.**001**
	Plant origin × inoculum treatment	1, 272	1.39	0.239
	Soil inoculum origin × inoculum treatment	1, 272	22.95	**<0**.**001**
	Plant origin × soil inoculum origin × inoculum treatment	1, 272	30.29	**<0**.**001**
Number of leaves	Plant origin	1, 4	8.68	**0**.**038**
	Soil inoculum origin	1, 272	6.20	**0**.**013**
	Inoculum treatment	1, 272	125.76	**<0**.**001**
	Initial plant height	1, 276	4.57	**0**.**034**
	Plant origin × soil inoculum origin	1, 272	5.36	**0**.**021**
	Plant origin × inoculum treatment	1, 272	0.10	0.756
	Soil inoculum origin × inoculum treatment	1, 272	9.98	**0**.**002**
	Plant origin × soil inoculum origin × inoculum treatment	1, 272	12.10	**<0**.**001**
Number of leaflets	Plant origin	1, 5	43.22	**0**.**002**
	Soil inoculum origin	1, 272	0.44	0.509
	Inoculum treatment	1, 272	44.89	**<0**.**001**
	Initial plant height	1, 269	2.25	0.135
	Plant origin × soil inoculum origin	1, 272	3.15	0.077
	Plant origin × inoculum treatment	1, 272	0.03	0.860
	Soil inoculum origin × inoculum treatment	1, 272	3.35	0.068
	Plant origin × soil inoculum origin × inoculum treatment	1, 272	13.05	**<0**.**001**
Chlorophyll concentration	Plant origin	1, 6	17.34	**0**.**005**
	Soil inoculum origin	1, 270	37.50	**<0**.**001**
	Inoculum treatment	1, 268	289.39	**<0**.**001**
	Initial plant height	1, 110	1.18	0.279
	Plant origin × soil inoculum origin	1, 268	13.34	**<0**.**001**
	Plant origin × inoculum treatment	1, 268	0.69	0.406
	Soil inoculum origin × inoculum treatment	1, 268	21.75	**<0**.**001**
	Plant origin × soil inoculum origin × inoculum treatment	1, 268	31.78	**<0**.**001**
Root:shoot ratio	Plant origin	1, 5	0.02	0.903
	Soil inoculum origin	1, 272	0.0038	0.951
	Inoculum treatment	1, 272	192.08	**<0**.**001**
	Initial plant height	1, 250	0.48	0.488
	Plant origin × soil inoculum origin	1, 272	9.18	**0**.**003**
	Plant origin × inoculum treatment	1, 272	0.73	0.394
	Soil inoculum origin × inoculum treatment	1, 272	4.29	**0**.**039**
	Plant origin × soil inoculum origin × inoculum treatment	1, 272	0.10	0.751
Number of nodules	Plant origin	1, NA	13.65	**<0**.**001**
	Soil inoculum origin	1, NA	7.57	**0**.**006**
	Initial plant height	1, NA	1.18	0.277
	Plant origin × soil inoculum origin	1, NA	8.37	**0**.**004**
Nodule activity	Plant origin	1, NA	2.06	0.151
	Soil inoculum origin	1, NA	25.91	**<0**.**001**
	Initial plant height	1, NA	0.59	0.443
	Plant origin × soil inoculum origin	1, NA	16.77	**<0**.**001**

**Fig. 2.**
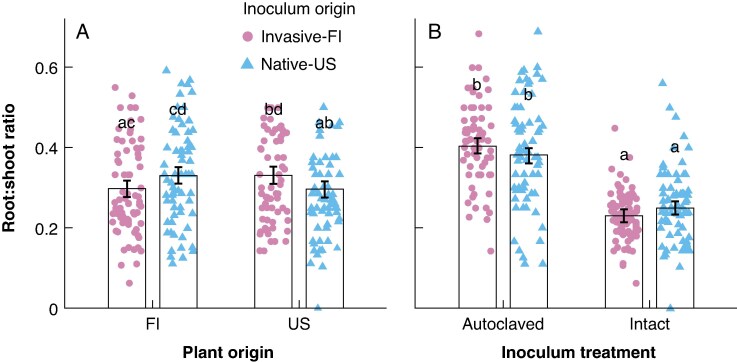
Root:shoot ratio of *Lupinus polyphyllus* in relation to (A) soil inoculum origin (FI, US) and plant origin (invasive [FI], native [US]), and (B) soil inoculum origin and soil inoculum treatments (autoclaved, intact) (mean ± standard error). Points represent raw data points. Statistical differences are shown with different letters (*P* < 0.05, Tukey’s test or a linear model).

For plants of both origins, treatment with the foreign soil inoculum resulted in ∼11 % higher root:shoot ratio (plant origin × soil inoculum origin; Table 1, Fig. 2A). Moreover, root:shoot ratio was higher in the autoclaved soil inoculum treatment than in the intact soil inoculum treatment for both soil inoculum origins, but in FI soil the effect was larger (FI soil, +76.9 %; US soil, +52 %; soil inoculum origin × inoculum treatment; Table 1, Fig. 2B).

**Fig. 3.**
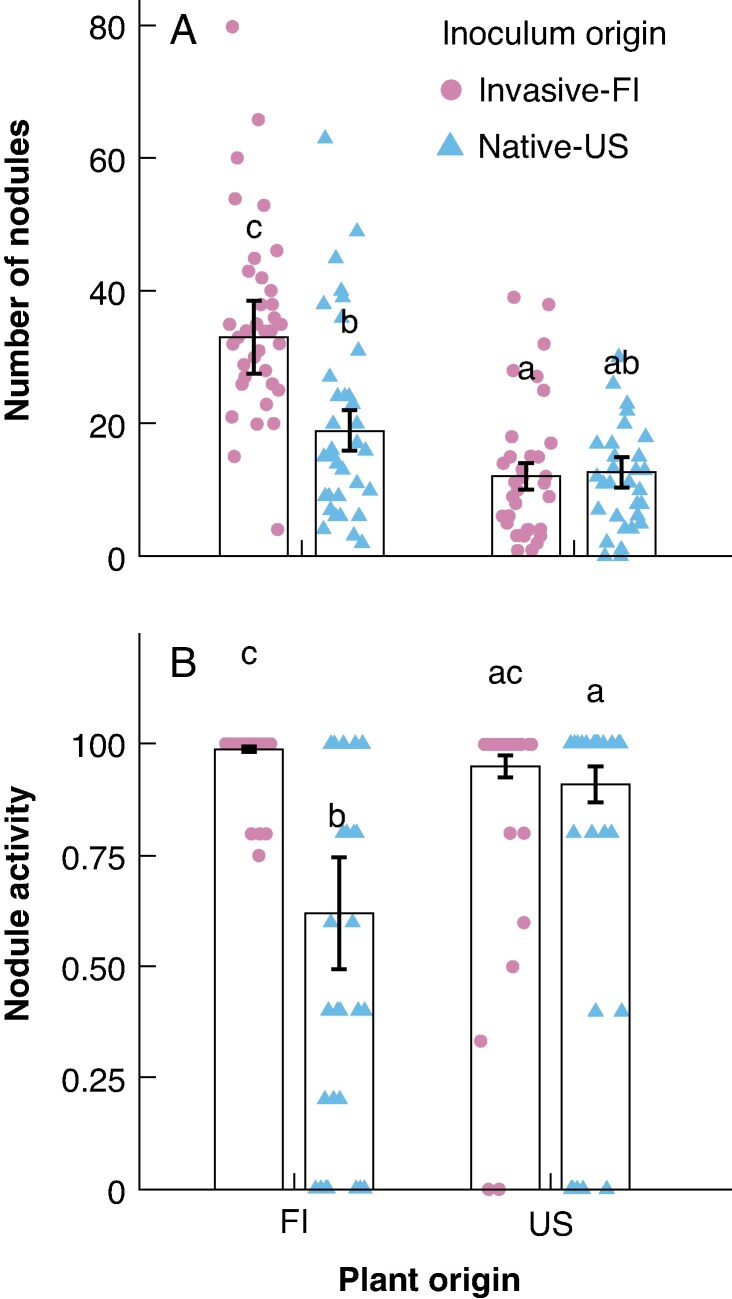
Effect of plant origin (invasive [FI] and native [US]) on (A) the number of nodules and (B) nodule activity of *Lupinus polyphyllus* grown with invasive (FI) and native (US) soil inocula (mean ± standard error). Points represent raw data points. Statistical differences are shown with different letters (*P* < 0.05, Tukey’s test or a linear model).

Nodule number and nodule activity were higher for FI plants that were grown in the FI soil inoculum treatment than in the US soil inoculum treatment (75.1 and 56.9 %, respectively), but these variables did not differ between soil inoculum origins for US plants (plant origin × soil inoculum origin; Table 1, Fig. 3). In the US soil inoculum treatment, FI plants produced 32 % fewer active nodules than US plants (Table 1, Fig. 3B).

### Allelopathy

*Taraxacum* seedlings grown in a substrate exposed to *L. polyphyllus* were larger in mean diameter than those grown in a control substrate with no history of the plant invader (mean ± s.e. = 22.5 ± 0.8 and 19.0 ± 1.5 mm, respectively; Table 2). Previous exposure to *L. polyphyllus* did not affect the number of *Taraxacum* seedlings that germinated (Table 2). Neither the number nor the diameter of *Taraxacum* seedlings showed a response to plant origin or soil inoculum treatment (Table 2).

**Table 2.** Results from linear fixed-effect models used to investigate the allelopathic effect of *Lupinus polyphyllus* on a local perennial herb (*Taraxacum* sp.). Two separate models were conducted for both response variables. Significant *P*-values (*P* < 0.05) are shown in bold; d.f. and d.d.f. denote the degrees of freedom in the numerator and denominator.

Response variable		Explanatory variable	d.f., d.d.f.	*F*	*P*
Number of seedlings	Model 1	Exposure to *L. polyphyllus*	1, 196	0.02	0.887
	Model 2	Plant origin (FI, US)	1, 139	1.66	0.199
		Inoculum treatment (autoclaved, intact)	1, 139	1.70	0.195
		Plant origin × inoculum treatment	1, 139	0.001	0.972
Seedling diameter (mm)	Model 1	Exposure to *L. polyphyllus*	1, 179	5.50	**0**.**020**
	Model 2	Plant origin	1, 127	0.09	0.760
		Inoculum treatment	1, 127	0.82	0.368
		Plant origin × inoculum treatment	1, 127	0.35	0.557

### Below-ground bacterial communities

In the soil inocula collected from the invasive (FI) and native (US) ranges, Chitinophagaceae was the most common bacterial family at all six sites (7–12 %) except for one invasive (FI) site, where bacteria in the family Bradyrhizobiaceae were most abundant (11 %; Supplementary Data Fig. S2).

Growing plants with soil inocula from the invasive and native ranges resulted in minor differences in the below-ground bacterial communities; inoculum origin explained only 2.5 % of the variation (Supplementary Data Table S2). For plants grown with the US soil inoculum, the bacterial communities were more heterogeneous than for those grown with the FI soil inoculum (Supplementary Data Fig. S3). The below-ground bacterial communities differed greatly between the autoclaved and intact soil inoculum treatments (Supplementary Data Table S2), with autoclaving reducing variation in the communities (Supplementary Data Fig. S3). Soil inoculum treatment also tended to affect the bacterial communities differently depending on plant and soil inoculum origins (Supplementary Data Table S2), but the explanatory power of these interactions was low (<2.5 %) compared with that of inoculum treatment (13 %). Most common bacterial families for plants grown in both the autoclaved and intact inoculum treatments were Comamonadaceae, Pseudomonadaceae and Chitinophagaceae (Supplementary Data Fig. S4).

## DISCUSSION

We investigated how inoculation with soil microbes from invasive (FI) and native (US) populations of the perennial legume *Lupinus polyphyllus* affects the performance of individual plants from both ranges. For plants of both origins, we found strong positive effects of soil microbial inoculation on all seven performance traits investigated. However, the soil microbiota from invasive (FI) sites had a stronger positive effect on the performance of plants of invasive (FI) origin than the soil microbiota from native (US) sites, whereas for native (US) plants the increase in performance induced by the soil microbiota was similar regardless of the origin of the soil inoculum (total biomass, number of leaves, number of leaflets and chlorophyll concentration). Differences in the composition of the below-ground bacterial communities between plants grown in soil inocula from invasive and native sites at the family level were minor.

### Greater performance of invasive plants with soil microbes from their own range

There are multiple possible explanations for the success of invasive (FI) plants of *L. polyphyllus* grown with the soil inoculum from their own range. A possible explanation is provided by the enhanced mutualist hypothesis. According to this hypothesis, soil collected from invasive sites may contain soil mutualists that have stronger positive effects on the performance of plants of invasive origin than the soil mutualists in native soils (Reinhart and Callaway, 2006). This idea is consistent with two of our main findings: the net positive effect of soil microbiota on the performance of invasive (FI) plants and the larger positive effect of the invasive (FI) soil inoculum compared with the native (US) soil inoculum. The nodule number and nodule activity of FI plants were 75 and 57 % higher, respectively, when they were inoculated with invasive (FI) soil compared with native (US) soil, which probably explains their greater success. Our result is also in line with a previous study by Sirivat *et al.* (2023), who found that non-native soil mutualists improved the performance of *L. polyphyllus* of invasive origin and suggested that these mutualists contributed to the invasion success of the species. Plants from native populations of *L. polyphyllus*, instead, are facilitated equally well by microbiota from both the invasive and native populations (this study; Kalske *et al.*, 2022). Thus, it seems that native (US) populations are able to benefit from non-native soil microbiota as well. Still, nodule activity was similar between FI and US plants grown in the invasive (FI) soil inoculum treatment. Invasive plant species tend to benefit more from soil microbiota from their own range because they have evolved to associate with local soil mutualists (Porter *et al.*, 2011). Thus, it is also possible that invasive (FI) populations have evolved to associate with the available rhizobia in the invasive (FI) range and ultimately form more effective associations with them than with native rhizobia (Shelby *et al.*, 2016). Alternatively, greater performance of invasive plants could be due to their ability to better exploit resources with local microbes or evolutionary shifts in their resource allocation to growth, as proposed by the evolution of increased competitive ability (EICA) hypothesis (Blossey and Nötzold, 1995). A previous common garden study on *L. polyphyllus* has also revealed the larger size of invasive (FI) than native (US) plants (but lower flowering probability and number of flowering shoots) when grown in a commercial soil (Ramula and Kalske, 2020). However, in the case of a shift in resource allocation, we would have expected that the autoclaved soil inoculum treatment would have also increased the growth of invasive plants compared with that of natives.

Another but perhaps less likely explanation for the greater success of invasive (FI) plants grown with the soil inoculum from their own range compared with that from the native range might be that invasive populations have escaped from harmful native soil microbes, or that soil pathogens may be less virulent in the invasive range (Dawson and Schrama, 2016). As an example, a study on six European forbs that are invasive in North America revealed that native soil suppressed their performance, while soil collected from the introduced range did not cause significant negative feedback on any of the species, likely because it did not contain native soil pathogens (Maron *et al.*, 2014). Here, plants of native (US) origin produced more leaves and had a higher leaf chlorophyll concentration than invasive (FI) plants when both were inoculated with soil collected from the native range. This supports our prediction that native (US) soil inoculum would increase the performance of native (US) plants more than that of invasive (FI) plants. The result could be explained by the presence of specialist native soil pathogens that affect invasive (FI) plants more negatively than native (US) plants, as the resistance of the former to soil pathogens from the native range might have been lost in the absence of such pathogens in the invasive range. However, contrary to the expectations of the enemy release hypothesis, the intact native (US) soil inoculum still had a net positive effect on invasive (FI) plants: they produced more biomass, had more leaves and had a higher leaf chlorophyll concentration than plants grown in the autoclaved soil inoculum treatment. This finding suggests that native (US) soil did not contain soil pathogens that suppressed the performance of plants of invasive (FI) origin or, if it does, that their negative effect was overridden by that of beneficial soil mutualists. It is also possible that invasive plants have adapted to perform well, despite the presence of pathogens. Differences in the traits between plants grown in the native and invasive soil inoculum treatments might have been related to differences in soil chemistry, which was not recorded in the present study. Given the fact that we used only a small amount of a diluted soil slurry (10 mL per 1-L pot), this explanation seems unlikely. Finally, different storage times of US and FI soils might have also contributed to the observed differences in plant performance in relation to the soil inoculum origin through e.g. changes in microbial densities. Nevertheless, we consider this possibility hypothetical because microbial activity tests conducted before the inoculation experiment revealed microbial growth in each soil inoculum source, confirming that the intact soil inocula of both origins contained living microbes (mean cover ± s.e.: intact US 76.3 ± 8.8 % and intact FI 86.7 ± 4.2 %; Supplementary Data Methods S1). Moreover, the below-ground bacterial communities showed only modest differences between soil inoculum origins at the end of the greenhouse experiment, but clearly differed between autoclaved and intact soil inoculum treatments (Supplementary Data Fig. S4). To distinguish between the alternative, not necessarily mutually exclusive, mechanisms for the greater performance of invasive plants grown with soil inocula from their own range requires more detailed data on soil microbiota. For example, sequencing the nodules of inoculated plants would enable characterization of putative pathogens and putative rhizobia, including potential shifts in their relative abundances between invasive and native origins.

We found that plants of both origins exhibited a similar resource allocation pattern. They had a higher root:shoot ratio when they were inoculated with foreign soil, which means that they allocated more biomass into roots and less biomass into shoots. Higher investment in roots by invasive (FI) plants grown in the native (US) soil inoculum treatment may be linked to their lower nodule activity in US soil. However, a similar relationship between nodule activity and root:shoot ratio was not detected for native (US) plants. In all plants, the autoclaved soil inoculum was associated with increased allocation to below-ground biomass. In this case, the absence of soil mutualists may have reduced the amount of nitrogen available for plants in the substrate, and, in response, plants may have allocated more resources to root systems to improve nutrient acquisition (Concha and Doerner, 2020).

### Lack of allelopathy

We did not observe allelopathy related to the plant invader in the present study. Substrate that had been previously occupied by *L. polyphyllus* had no effect on the germination of *Taraxacum* seedlings regardless of plant origin (invasive vs native). In other words, invasive FI plants did not differ from native US plants in terms of allelopathy. Similarly, autoclaving treatment had no effect on the germination or seedling size of *Taraxacum*. Overall, these findings suggest that allelopathy by soil conditioning may not be an important mechanism for allelopathy in *L. polyphyllus*. A recent study reported that, when seeds of co-occurring native herbs were placed in Petri dishes with shoot and root leachates from invasive populations of *L. polyphyllus*, germination was strongly suppressed (Kalske *et al.*, 2023). The lack of allelopathic effects observed in the present study could be due to differences in the experimental set-up compared with previous studies. Leachates from above-ground plant tissues have stronger allelopathic effects than leachates prepared from below-ground parts of the plants (Zhang *et al.*, 2021; Kalske *et al.*, 2023), which could indicate that allelopathy through soil alone might be less effective than that from above-ground plant parts (Zhang *et al.*, 2021). Moreover, growth in a substrate resembles more realistic natural conditions than that in Petri dishes, and may result in weaker allelopathic effects as allelochemicals tend to accumulate in Petri dishes due to a lack of drainage (Álvarez-Iglesias *et al.*, 2014). Despite no difference in germination, the mean diameter of *Taraxacum* seedlings was larger in a substrate that had been exposed to *L. polyphyllus*, which could have been due to an increase in nitrogen content and mobilization of phosphorus in *Lupinus*-exposed soils (Lambers *et al.*, 2013; Hanslin and Kollmann, 2016). However, it is also possible that these results were due to fertilization during the initial greenhouse experiment where pots were fertilized twice with nutrient solution. Without chemistry data from the substrate in the common garden experiment, we cannot distinguish between these two alternatives.

### Minor differences in below-ground bacterial communities between soil inoculum origins

Soil microbiota, irrespective of its origin, had a net positive effect on *L. polyphyllus* plants of both origins because the intact soil inoculum treatment typically increased plant performance more than inoculation with the autoclaved soil inoculum. Characterization of the field soil bacterial communities showed that the two most common bacterial families in the soils collected from three invasive (FI) and three native (US) sites of *L. polyphyllus* were Chitinophagaceae and Bradyrhizobiaceae, although the sample size was limited. A previous study based on a more comprehensive sampling has also reported that these are indeed the most abundant bacterial families in Finnish soils invaded by the study species (Mousavi and Ramula, 2024). Earlier studies have described that some members of the family Chitinophagaceae can protect plants against fungal root disease and promote plant growth (Madhaiyan *et al.*, 2015; Carrión *et al.*, 2019). The family Bradyrhizobiaceae, in turn, includes the genus *Bradyrhizobium*, which is known to be the most widespread and dominant group of rhizobia nodulating *L. polyphyllus* (Stępkowski *et al.*, 2018; Ramula *et al.*, 2023). Recent evidence has underlined the importance of soil mutualists for the performance of *L. polyphyllus* (Sirivat *et al.*, 2023), and its ability to benefit from soil microbiota collected from different locations is likely to be an important factor in its invasion success (Kalske *et al.*, 2022).

In the greenhouse experiment, plant and soil inoculum origins (FI, US) had a minor effect on the below-ground bacterial communities at the family level that were largely shaped by inoculum treatment (autoclaved, intact). Autoclaving homogenized bacterial communities, although the top three bacterial families (Comamonadaceae, Pseudomonadaceae, Chitinophagaceae) were present in pots receiving autoclaved and intact soil inocula. All of these three families are common in soils and are involved in nutrient cycling or the degradation of organic matter (e.g. Wolińska *et al.*, 2018). Our findings indicate that the trait differences observed between plants grown in soil inocula obtained from invasive and native sites may not be driven by bacterial families that are most abundant. A visual inspection of different genera within the family Bradyrhizobiaceae did not reveal major differences in relative abundances between soil inoculum origins, with the most abundant bacterial genus being *Bradyrhizobium* (i.e. rhizobia) in the intact inoculum treatment for both soil origins (Supplementary Data Fig. S5). Putative pathogens within Bradyrhizobiaceae primarily belong to the genus *Afipia* (Marcondes de Souza *et al.*, 2014). In the present study, they were more abundant in the US soils, although their relative abundance was minimal (<0.03 %; Supplementary Data Fig. S5). Nevertheless, a functional approach using metatranscriptomics would be useful to provide more information on bacterial metabolic activity and function (Bailly *et al.*, 2007). It should be also noted that we focused on bacteria here and did not consider the below-ground fungal communities that can equally modify plant phenotype (e.g. Trivedi *et al.*, 2020).

### Conclusions

Here, the positive effects of soil microbes from invasive sites (FI) on the performance of *L. polyphyllus* from invasive populations are consistent with the enhanced mutualism hypothesis, but could also reflect other adaptations either in plants or soil microbiota, or in both. Regardless of the exact mechanism for the greater performance of invasive plants, the present study demonstrates that invasive and native plants interact differentially with their local soil microbiota. Although local soil mutualists were superior to invasive (FI) populations, it seems that native (US) populations of *L. polyphyllus* are not strongly adapted to local soil mutualists in the native range, but are able to associate with and benefit from soil mutualists outside their own range as well, which may partly explain the global invasion success of this species.

## Supplementary Material

mcag067_Supplementary_Data

## Data Availability

The raw data of bacterial samples were deposited in the NCBI Sequence Read Archive (SRA) database under the BioProject PRJNA1295829 (accession numbers SRR34701959–SRR34702239 and SRR34855551–SRR34855556). The data and R code used for the statistical analyses were deposited on Zenodo https://doi.org/10.5281/zenodo.16892097.

## SUPPLEMENTARY DATA

Supplementary data are available at *Annals of Botany* online and consist of the following. Table S1: locations of the study populations of the perennial *Lupinus polyphyllus* for collection of seeds and soil inocula. Table S2: results of PERMANOVA testing for differences in soil bacterial communities of *Lupinus polyphyllus* of different origins grown with intact and autoclaved soil inocula. Methods S1: microbial activity tests. Methods S2: bioinformatics. Figure S1: rarefaction curves of ASV numbers in relation to the number of sequences sampled of *Lupinus polyphyllus*. Figure S2: relative abundances of the six most common bacterial families in three native and three invasive sites of *Lupinus polyphyllus*. Figure S3: non-metric multidimensional scaling (NMDS) ordination and Venn diagram of the soil bacterial communities of *Lupinus polyphyllus* grown with intact and autoclaved soil inocula. Figure S4: relative abundances of the six most common bacterial families of *Lupinus polyphyllus* grown with intact and autoclaved soil inocula from invasive and native sites. Figure S5: relative abundances of eight bacterial genera within the family Bradyrhizobiaceae sampled from the rhizosphere of *Lupinus polyphyllus* grown with intact and autoclaved soil inocula from invasive and native sites.
