# Supplementary material for: Soil microbiota promote the success of the perennial legume Lupinus polyphyllus more strongly in invasive than in native populations
Source: Ann Bot. 2026 Mar 19;137(7):2257–67. doi: 10.1093/aob/mcag067 (PMC13319345; doi:10.1093/aob/mcag067)
Supplement: mcag067_Supplementary_Data [file mcag067_supplementary_data.docx]

## Supporting Information

**Table S1** Locations of the study populations of the perennial *Lupinus polyphyllus* for collection of seeds and soil inocula.

| Population | Country/region | Lat, Long |
| --- | --- | --- |
| US-1 | US/CA-Tahoe | 39.35, -120.35 |
| US-2 | US/CA-Sagehen Creek | 39.43, -120.24 |
| US-3 | US/OR-Winema | 42.46, -122.40 |
| FI-1 | FI/Turku | 60.43, 22.39 |
| FI-2 | FI/Turku | 60.49, 22.17 |
| FI-3 | FI/Turku | 60.51, 22.29 |

**Methods 1** Microbial activity tests.

We checked microbial activity in the growth medium as well as in the autoclaved and intact soil inoculants separately for each site following Trevors (1996), by observing microbial growth (total cover of microbial colonies per plate) on tryptone-yeast agar plates after a 9-day incubation at room temperature in May 2023. We had three plates per population per inoculum treatment (intact or autoclaved; altogether 6 populations × 2 treatments × 3 plates = 36 plates) and four plates for the growth medium. The total cover of microbial colonies in plates primed with the autoclaved soil inoculant was 76% lower than in those treated with intact soil (mean ± SE cover: autoclaved 5.1 ± 0.8%, intact 81.5 ± 3.6%). The microbial cover in the plates primed with the growth medium was intermediate to the two inoculum treatments (40.0 ± 12.7%), but this microbial background was the same for all experimental plants. Microbial cover was comparable for the intact soil inocula between regions (mean ± SE cover: intact US 76.3 ± 8.8%, intact FI 86.7 ± 4.2%), confirming that all soil inoculants contained living microbes in the beginning of the greenhouse experiment (mean cover for individual sites: 66-95%).

Reference:

Trevors JT 1996. Sterilization and inhibition of microbial activity in soil. *Journal of Microbiological Methods* **26**: 53–59.

**Methods S2** Bioinformatics.

*Microbial samples*

We characterised the bacterial communities of the soil inocula collected from six sites, based on a pooled soil sample per site. These samples were taken from the soils stored in the cold room (+4°C) right before the beginning of the greenhouse experiment (about three months for the US soils and one month for the FI soils). Moreover, we sampled a substrate from 288 pots in the greenhouse experiment (evenly distributed between seed origins (6 populations) and soil inoculants (6 sites)) at the end of the experiment only. All the samples were kept at -20°C until DNA extraction that took place in the summers of 2023 and 2024 for the field soil and greenhouse samples, respectively, by following the same protocol described below.

*DNA extraction and PCR amplification*

The methods for amplicon library preparation targeting the 16S rRNA gene (V4 region) were adapted from those developed for the Earth Microbiome Project (<https://earthmicrobiome.org/>). In short, the PCR protocol consisted of initial denaturation at 94°C for 4 min, then 30 cycles of 94°C for 20 s, 57.5°C for 15 s, and 68°C for 30 s, using the barcoded V4 primer set 515F/806R (Parada et al. 2016) and Platinum II Taq Hot-Start (Invitrogen™, Thermo Fisher Scientific, Waltham, Massachusetts, USA). Replicated PCR products were pooled into DNA libraries and then purified and size selected with a SPRI bead clean-up following Vesterinen et al. (2016). Prepared libraries were sequenced using Illumina Miseq v2 sequencing (2 × 250 bp) at the Finnish Functional Genomic Centre in Turku, Finland. Each library contained a negative control (one for the field soil samples and three in total for the greenhouse samples).

*Bioinformatics*

We processed a total of 985 679 and 26 663 753 raw reads for field and greenhouse soil samples, respectively by applying the ASV-based DADA2 pipeline v.1.24.0 (Callahan et al. 2016) and following the online DADA2 Pipeline Tutorial (1.16) (<https://benjjneb.github.io/dada2/tutorial.html>) for sequences. After filtering, denoising, merging, and removing chimers, 702 912 sequences from six field samples and 18 406 463 sequences from 288 greenhouse samples remained for further analyses. We assigned taxonomic identities to the sequences using the Ribosomal Database Project’s (RDP) training set 19 (updated August 2023), formatted for use with DADA2, as the 16S rRNA reference dataset. Initially, we identified 9 231 and 45 392 amplicon sequence variants (ASVs) across the field and greenhouse samples, respectively. The ASV and taxonomy tables were imported to RStudio (2024.12.1), which is based on R version 4.2.0 (R Core Team 2022). We first removed all singletons using the phyloseq package (McMurdie & Holmes, 2013) and non-bacterial ASVs using the microeco package (Liu et al., 2021). We rarefied the samples (to 10 000 and 58 114 sequences per sample for the field and greenhouse soil samples, respectively) using the microeco package (Liu et al., 2021) and converted absolute abundances into relative abundances. For the six field soil samples, we retrieved 8 977 bacterial ASVs, which were assigned to 30 phyla, 74 classes, 135 orders, 233 families, and 542 genera. For the greenhouse samples (n=264), we retrieved 21 142 bacterial ASVs, which were assigned to 33 phyla, 79 classes, 148 orders, 278 families, and 768 genera.

References:

Callahan BJ, McMurdie PJ, Rosen MJ, Han AW, Johnson AJA, Holmes SP. 2016. DADA2: High-resolution sample inference from Illumina amplicon data. *Nature Methods* **13**: 581–3.

Liu C, Cui Y, Li X, Yao M. 2021. microeco: An R package for data mining in microbial community ecology. *FEMS Microbiology Ecology*. **97**: fiaa255. Available from: <https://doi.org/10.1093/femsec/fiaa255>

McMurdie PJ, Holmes S. 2013. phyloseq: an R package for reproducible interactive analysis and graphics of microbiome census data. *PloS One* **8** :e61217.

Parada AE, Needham DE, Fuhrman JA. 2016. Every base matters: assessing small subunit rRNA primers for marine microbiomes with mock communities, time series and global field samples. *Environmental Microbiology* **18**: 1403-1414.

Vesterinen EJ, Ruokolainen L, Wahlberg N, Peña C, Roslin T, Laine VN, Vasko V, Sääksjärvi IE, Norrdahl K, Lilley TM. 2016. What you need is what you eat? Prey selection by the bat *Myotis daubentoniid*. *Molecular Ecology* **25**: 1581–1594.


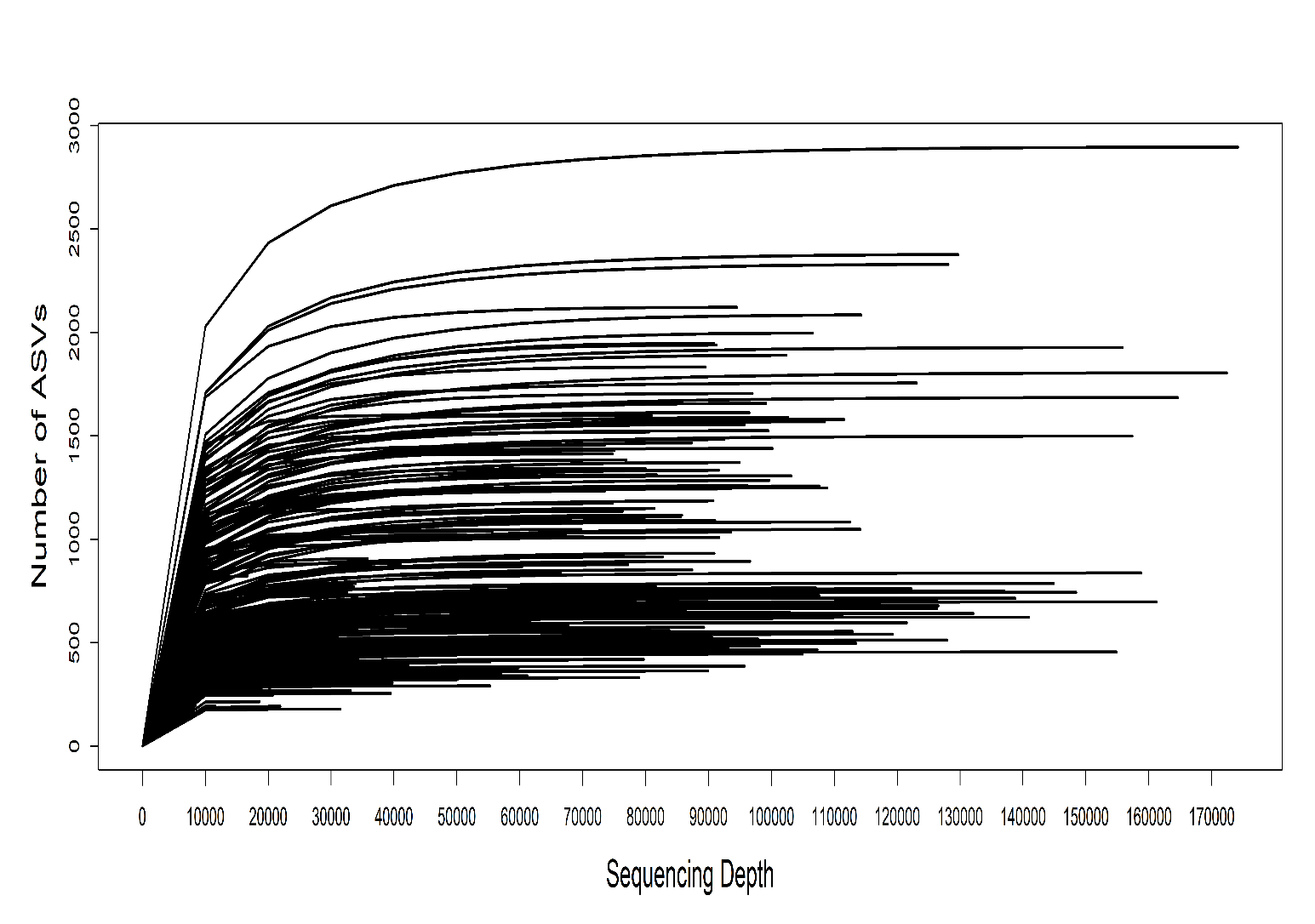


**Figure S1** Rarefaction curves of ASV numbers in relation to the number of sequences sampled of *Lupinus polyphyllus*.


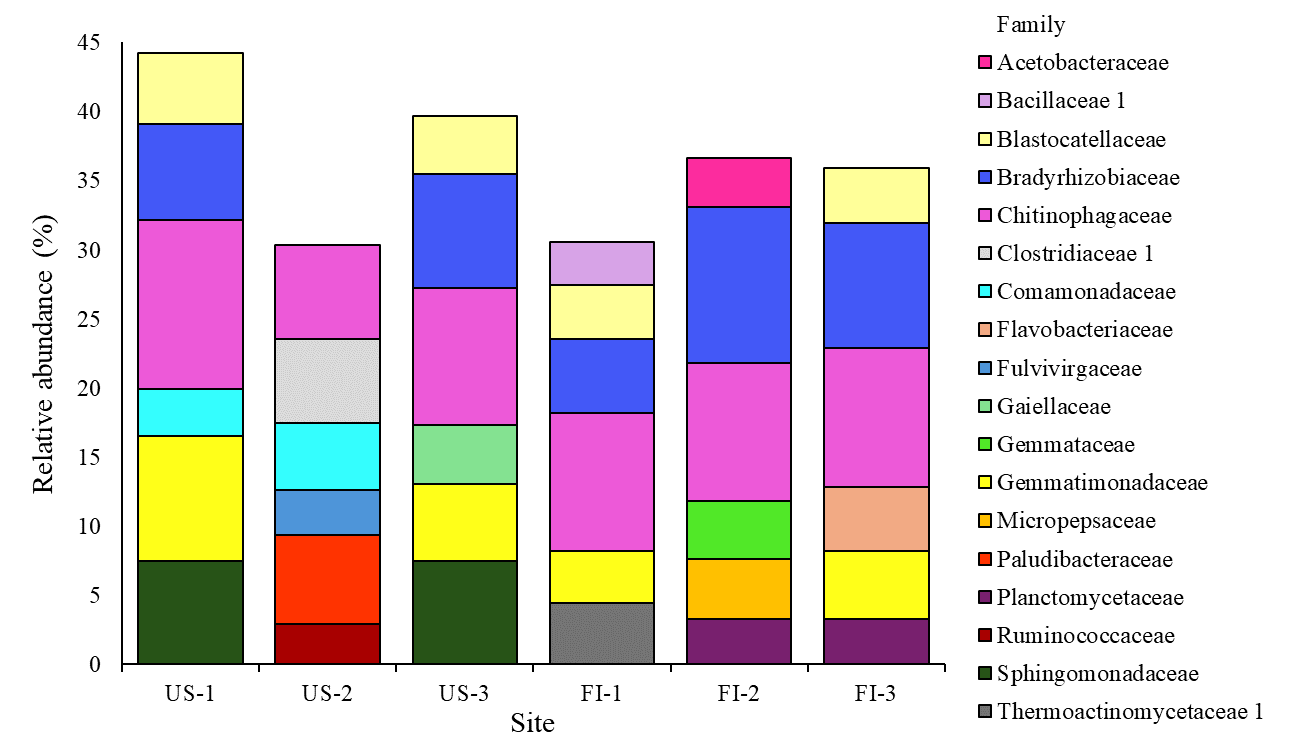


**Figure S2** Relative abundances of the six most common bacterial families in three native (US) and three invasive (FI) sites of *Lupinus polyphyllus*.

**Table S2** Results of PERMANOVA testing for differences in soil bacterial communities of *Lupinus polyphyllus* of different origins grown with intact and autoclaved soil inocula from invasive (FI) and native (US) sites. Analyses are based on Bray-Curtis dissimilarities calculated from the relative abundances of bacterial ASVs. *P*-values <0.05 are in bold.

| **Fixed factors** | **df** | ***F*** | ***R*^2^** | ***P*** |
| --- | --- | --- | --- | --- |
| Plant origin (FI, US) | 1 | 7.434 | 0.023 | **0.001** |
| Soil inoculum origin (FI, US) | 1 | 8.303 | 0.025 | **0.001** |
| Inoculum treatment (intact, autoclaved) | 1 | 43.157 | 0.132 | **0.001** |
| Plant origin × soil inoculum origin | 1 | 1.048 | 0.003 | 0.341 |
| Plant origin × inoculum treatment | 1 | 2.383 | 0.007 | **0.003** |
| Soil inoculum origin × inoculum treatment | 1 | 8.125 | 0.025 | **0.001** |
| Plant origin × soil inoculum origin × inoculum treatment | 1 | 0.884 | 0.003 | 0.616 |

b

**Figure S3** Non-metric multidimensional scaling (NMDS) ordination of the soil bacterial communities of the legume *Lupinus polyphyllus* grown with intact and autoclaved soil inocula from invasive (FI) and native (US) sites (a). Venn diagram showing the number of unique and shared ASVs with their relative abundances for plants grown with intact and autoclaved soil inocula (b).

**
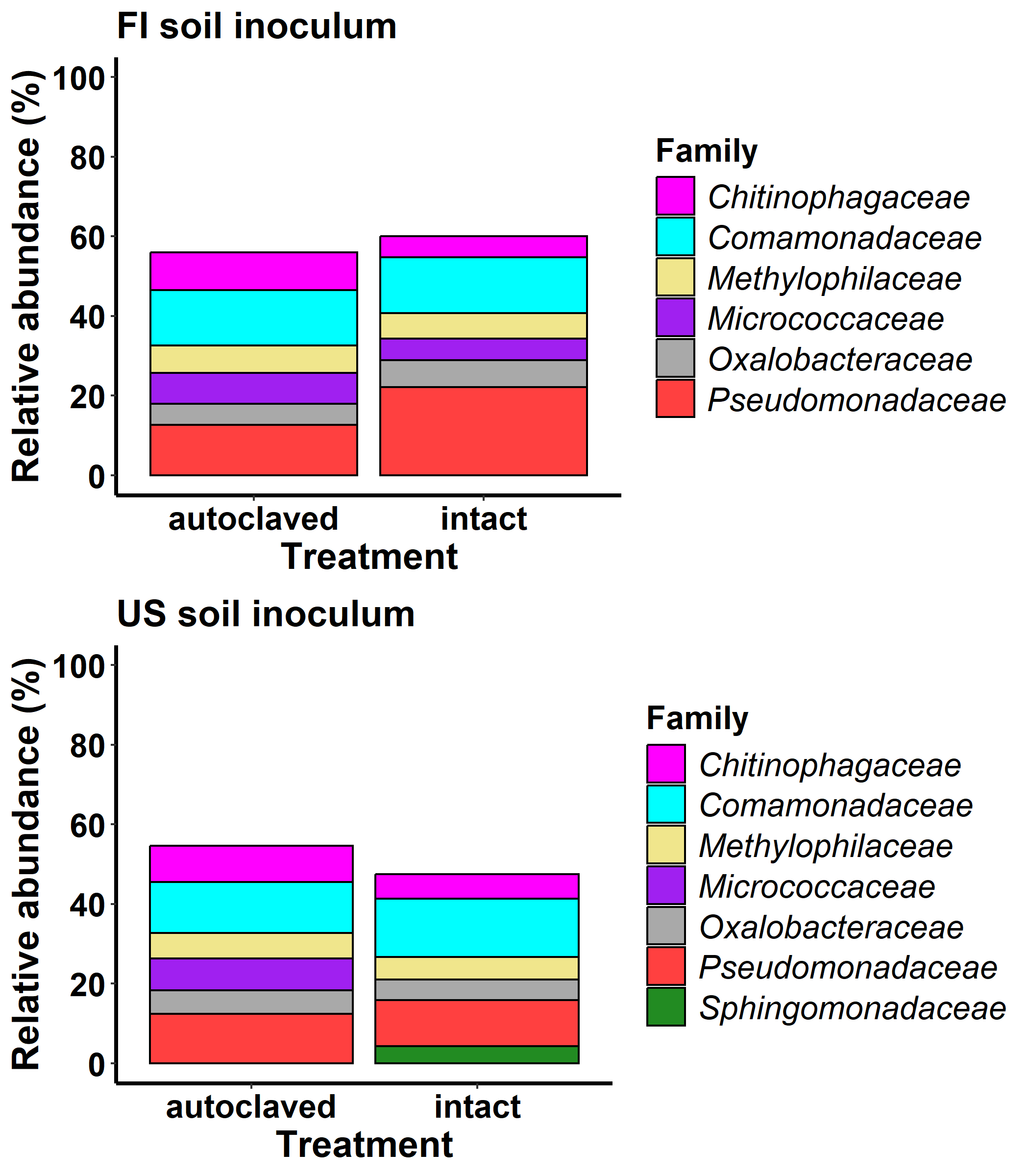
**

**Figure S4** Relative abundances of the six most common bacterial families of *Lupinus polyphyllus* grown with intact and autoclaved soil inocula from invasive (FI) and native (US) sites.

**Figure S5** Relative abundances of eight identified bacterial genera within the family Bradyrhizobiaceae sampled from the rhizosphere of *Lupinus polyphyllus* grown with intact and autoclaved soil inocula from invasive (FI) and native (US) sites.
